# Pregnenolone Reduces Stress-Induced Craving, Anxiety, and Autonomic Arousal in Individuals with Cocaine Use Disorder

**DOI:** 10.3390/biom12111593

**Published:** 2022-10-29

**Authors:** Verica Milivojevic, Lily Charron, Nia Fogelman, Gretchen Hermes, Rajita Sinha

**Affiliations:** The Yale Stress Center, Department of Psychiatry, Yale University School of Medicine, New Haven, CT 06519, USA

**Keywords:** neuroactive steroids, pregnenolone, cocaine use disorder, stress, craving, anxiety, heart rate

## Abstract

Chronic cocaine use leads to adaptations in stress biology and in neuroactive steroid system. These adaptations are associated with high cocaine craving and increased relapse risk. This study tested whether potentiation of the neuroactive steroid system with the precursor pregnenolone (PREG) affects stress- and cue-induced cocaine craving, anxiety and autonomic response in individuals with cocaine use disorder (CUD). Thirty treatment-seeking individuals (21 Male, 9 Female) with CUD were randomized to placebo (PBO) or supraphysiologic PREG doses of 300 mg or 500 mg per day for 8 weeks. After 2 weeks of treatment, participants were exposed to 5-min personalized guided imagery provocation of stress, cocaine, or neutral/relaxing cues in a 3-day experiment, one condition per day on separate days, in a random, counterbalanced order. Repeated assessment of cocaine craving, anxiety, heart rate (HR), systolic (SBP) and diastolic blood pressure (DBP) were assessed on each day. PREG significantly increased pregnenolone levels compared to PBO. Both PREG doses decreased stress- and cocaine cue-induced craving and reduced both stress- and cue-induced anxiety only in the 500 mg/day group. The 500 mg/day PREG group also displayed decreased stress-induced HR, SBP and DBP. Findings indicate that pregnenolone decreases stress- and cocaine cue-provoked craving and anxiety and reduces stress-induced autonomic arousal in individuals with CUD.

## 1. Introduction

Cocaine use disorder (CUD) is a common and serious health problem [1] for which there are currently no approved medications. Early abstinence from cocaine is marked by dysregulated stress- and cue-induced autonomic and emotional changes, and is significantly associated with increased drug craving, drug use and relapse risk [2,3,4,5,6]. This research has directly linked stress- and drug cue-induced craving, and their related disruption in stress biology to drug use and relapse risk, suggesting that treatments that reverse these processes may be useful targets in drug relapse prevention [7]. Importantly, disrupted physiologic and neuroendocrine responses in substance use disorders are associated with reductions in GABAergic control of the stress axis [8]. Recent basic science and clinical evidence indicate that GABAergic neuroactive steroids and their neuroactive precursor pregnenolone (PREG) normalize the stress response and reduce drug craving, anxiety, and drug intake [9,10,11,12], suggesting that PREG may be acting by normalizing stress system upregulation and potentially decreasing related compulsive drug seeking.

Both drugs of abuse and stress directly affect GABAergic transmission and neuroactive steroid levels, but direction varies as a function of acute effects or chronic adaptations [8,13,14]. Emerging evidence suggests that PREG-derived GABAergic neuroactive steroids may play a role in modulating negative reinforcement effects of drugs and also impact stress- and drug-cue associated relapse [13]. GABAergic neuroactive steroids are potent enhancers of GABA_A_ receptor function, and produce anxiolytic, anticonvulsant and hypnotic effects similar to those induced by other GABA_A_ receptor potentiating drugs [15,16]. Notably, fluctuations in peripheral and brain levels of the most widely studied GABAergic neuroactive steroid allopregnanolone (ALLO) have been associated with motivational mood-related changes during pregnancy, menopause, and psychiatric disorders, including depression, premenstrual dysphoric disorder, schizophrenia and bipolar disorder [17,18,19,20,21]. In addition, intraperitoneal (i.p.) injections of CRF and ACTH in rats increase brain and plasma levels of GABAergic neuroactive steroids [22]. These findings indicate that GABA potentiating neuroactive steroids may play a crucial compensatory role in mediating homeostasis in response to stress [8,23,24]. However, their effects on chronic cocaine-related allostatic neuroadaptations have rarely been assessed thus far.

Acute drug intake and acute stress increase GABAergic transmission and neuroactive steroids levels [10,22,25], and pharmacological challenges of the hypothalamic-pituitary-adrenal (HPA) axis potently increase levels of PREG [26]. Acute alcohol, for example, has been shown to increase GABAergic neuroactive steroids in plasma, cerebral cortex and hippocampus in rats [27,28,29,30]. On the other hand, chronic drug use and related neuroadaptations down-regulate GABAergic transmission [8] and decrease neuroactive steroid levels in the brain and periphery [10,14,28], arguing that increasing endogenous neuroactive steroids may improve drug-related outcomes. For example, chronic alcohol exposure reduced both plasma and brain levels of ALLO [28], and ALLO administration attenuated drug-primed reinstatement of cocaine [12] and cue-induced reinstatement [31] in cocaine dependent animals. Moreover, ALLO administration decreased cocaine primed reinstatement in female, but not in male rats [32], and decreased yohimbine-induced cocaine reinstatement in female rats only [33].

These preclinical findings suggest that GABAergic neuroactive steroids and their precursor pregnenolone may be acting by normalizing stress system upregulation and potentially decreasing related compulsive drug seeking, but whether pregnenolone would have beneficial effects in individuals with CUD has not been tested thus far. Therefore, we conducted a pilot experimental study in treatment engaged individuals with CUD who were part of an ongoing preliminary 8-week double-blind placebo-controlled trial of two supraphysiologic doses of pregnenolone (PREG; 300 mg/day, 500 mg/day) versus placebo (PBO). In week 2 of the trial, subjects participated in a 3-day laboratory experiment where on each day they self-administered the assigned study drug in the laboratory and this was followed by exposure to 5-min personalized guided imagery provocation of stress, alcohol, or active control neutral/relaxing cues, one condition per day on separate days, in a random, counterbalanced order, and their stress- and cocaine cue- provoked cocaine craving, subjective anxiety, and autonomic responses were assessed. On the basis of the previous research cited above, we hypothesized that pregnenolone doses will reduce stress- and cocaine cue- provoked craving and anxiety and normalize autonomic responses in treatment engaged individuals with CUD.

## 2. Materials and Methods

### 2.1. Participants

Thirty treatment-seeking individuals (21M/9F) with cocaine use disorder (CUD) participated in the study. All participants were recruited via local advertisements around the New Haven area. Current CUD criteria were determined using the Structured Clinical Interview for the Diagnostic and Statistical Manual of Mental Disorders 5 (SCID-5; [34]) and confirmed with positive urine toxicology screens collected during the initial eligibility assessment period. Exclusion criteria included: DSM-5 substance use disorder for any other psychoactive substance other than alcohol and nicotine, including opiate use disorder and including heroin (assessed and confirmed via urine toxicology screen in addition to SCID-5); any psychotic disorder or current Axis I psychiatric symptoms requiring specific attention; significant underlying medical conditions such as cerebral, renal, or cardiac pathology which in the opinion of study physician would preclude patient from fully cooperating or be of potential harm during the course of the study. All individuals underwent stringent medical assessments including electrocardiography and laboratory tests of renal, hepatic, pancreatic, hematopoietic and thyroid function, and a physical exam conducted by the study physician to determine study eligibility. Written and verbal consent was obtained from all participants and the Human Investigation Committee of the Yale University School of Medicine approved the study.

### 2.2. Study Procedures

Upon determination of eligibility, participants were randomized to receive daily placebo (PBO) or one of two doses of pregnenolone, 300 mg/day or 500 mg/day (oral administration, in b.i.d. dosing) in a double-blind manner for a total period of 8 weeks. For the current experimental study, scripts for the individualized guided imagery induction were developed (see below) using the well-established standardized procedures, as described in previous studies [35,36] during week 1 of their treatment. In week 2, a 3-day laboratory experiment involving participation in 3 separate experimental testing sessions on 3 separate days was conducted. Research staff were blind to order of imagery condition presented per day and subjects also remained blind until imagery presentation. Order of imagery condition was randomized and counterbalanced across subjects.

### 2.3. Study Medication Dosing and Compliance/Adherence

Identical pregnenolone (150 mg and 250 mg strength) and placebo capsules that also contained 25 mg of Riboflavin were formulated by the Yale University research pharmacist (Investigational Drug Services, IDS) and prepared for dispensing in 1-week bottles. Study participants, investigators and research staff were blind to the medication condition. Medication randomization was conducted by the Yale Stress Center Biostatistician and randomization was balanced for age, sex, smoking status, CUD severity, and education. The Biostatistician also provided de-identified dummy subject IDs for the Medication group so that the current analyses could be conducted in a blinded manner. This laboratory study was part of a larger study period of 8 weeks, where the study medication was provided in 1-week bottles for self-administration at home in the morning and evening at 8 a.m. and 8 p.m. during the 8-week trial. Importantly, in week 2 on laboratory experiment days, research participants were told not to take their 8 a.m. dose, and instead brought their bottle of medication to the laboratory and self-administered the morning dose at 1 p.m. in front of research staff before the experiment to ensure medication compliance for the current experiment (see below).

Medication Adherence: Compliance with daily dosing was monitored using (a) riboflavin detection in weekly urine provided by participants in the clinic, (b) using the smartphone-based video monitoring tool eMocha Mobile Health, Inc. (Baltimore, MD, USA), and (c) blood levels of pregnenolone collected on each of the three laboratory experimental days. Furthermore, on each laboratory session day, participants took the morning medication upon arrival at 1:00 p.m. and immediately before lunch, directly in front of study staff as a critical step to ensure medication adherence and acute medication effect on experimental days.

### 2.4. Imagery Script Development Procedures

Imagery script development was conducted in week 1 in a session prior to the laboratory experiment. Procedures are based on methods developed by Lang and his colleagues [37], and further adapted and validated in our previous studies [3,36,38,39,40]. Briefly, the stress imagery script was based on subjects’ descriptions of a recent “most stressful” adverse personal event that made them “sad, mad or upset”, that they were not able to control in the moment. “Most stressful” was determined by having the subjects rate their perceived stress on a 10-point Likert scale where 0 = not at all stressful and 10 = the most stress they felt recently in their life. Only situations rated as 8 or above were accepted as appropriate for script development (e.g., being fired from their job, marital conflict situation). The cocaine-related cue scripts were developed by having subjects identify a recent situation that included cocaine-related stimuli and resulted in subsequent cocaine use. Cocaine-related situations that were associated with negative affect or psychological distress were not allowed. A relaxing, non-physiologically arousing and non-cocaine related script was developed from the subjects’ description of a personal, relaxing situation (e.g., being at the beach; fall afternoon reading at the park). In addition to the script development, on the day of the first laboratory session, subjects were brought into the testing room in order to acclimatize them to specific aspects of the study procedures including the subjective rating forms and training in relaxation and imagery procedures, as previously described in [36].

### 2.5. Laboratory Sessions (Conducted across Three Separate Days)

**Figure 1:**
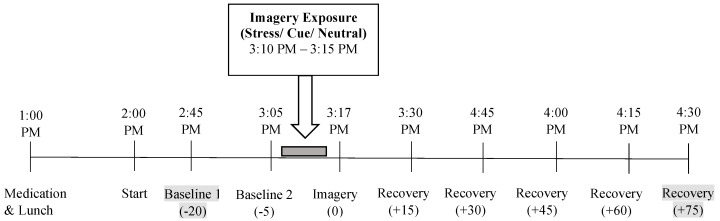
Schematic illustration of the laboratory sessions conducted on separate days, with order of imagery condition randomized and counter balanced. Participants self-administered study medication at 1:00 p.m. on each day prior to the experimental sessions. Cocaine craving and anxiety ratings, heart rate (HR), and systolic (SBP) and diastolic blood pressure (DBP), were collected at baseline (−20 and −5 min prior to imagery), immediately following imagery presentation (0) and every 15 min after the imagery period, up to 75 min (+15, +30, +45, +60, +75). Blood samples, highlighted in grey, to assess pregnenolone levels were collected at baseline (−20 min) and at the last timepoint (+75 min).

Participants were instructed to abstain from using cocaine after midnight prior to the laboratory sessions. Female subjects completed the laboratory sessions during the follicular phase of their menstrual cycle when both progesterone and estradiol levels are low and remain stable. Participants were brought into the testing room at 1:30 p.m., after a standard lunch provided at 1:00 p.m. after self-administration of study drug. Patients who were smokers were allowed a smoke break immediately prior to 1:30 p.m. in order to reduce potential nicotine withdrawal during the session. After settling in a sitting position on a reclining chair in an experimental testing room, a heparin-treated intravenous (IV) catheter was inserted at 2:00 p.m. by the research nurse in the antecubital region of the subject’s non-preferred arm in order to periodically obtain blood samples. A Critikon Dinamap 120 Patient Monitor was also placed on the subject’s preferred arm, including a pulse sensor which was placed on the subject’s forefinger. This was followed by a 40-min adaptation period during which the subjects were provided relaxation instructions to ensure stable psychophysiological state prior to each lab day. Immediately following the adaptation period, subjects were provided headphones and given the following instructions for the 5-min imagery procedure: “Close your eyes and imagine the situation being described, ‘as if’ it were happening right now. Let your body and mind get completely involved in the situation, doing what you would do in the real situation”. Cocaine craving and subjective emotion ratings, heart rate (HR), and systolic (SBP) and diastolic blood pressure (DBP were collected at baseline (−20 and −5 min prior to imagery), immediately following imagery presentation (0) and every 15 min after the imagery period, up to 75 min (+15, +30, +45, +60, +75), see Figure 1. Blood samples for assessment of circulating pregnenolone levels were collected at the −20 min baseline timepoint and the +75 min after imagery timepoint.

### 2.6. Laboratory Assessments

Cocaine Craving: Cocaine craving was assessed using the Cocaine Craving Questionnaire (CCQ)-NOW, a brief well validated 10-item self-report craving scale [41].

Anxiety: Study participants were asked to rate how tense, anxious, and/or jittery they feel using a 10-point visual analog scale (VAS) in which 0 = “not at all” and 10 = “more than ever”.

Heart Rate and Blood Pressure: A Critikon Dinamap 120 Patient Monitor (GE Medical Systems, Tampa, FL, USA) was used to assess heart rate, systolic blood pressure (SBP) and diastolic blood pressure (DBP) at the specific timepoints outlined above in the Laboratory Sessions section.

Pregnenolone Levels: Baseline (−20 timepoint before imagery) and recovery time point (+75 timepoints after imagery) blood samples were collected on each laboratory day to measure plasma pregnenolone levels. All tubes were placed on ice immediately after drawing, and then aliquoted after being centrifuged at 4 °C within 30 min of collection. Samples were then stored at −80 °C until processing at the Yale Center for Clinical Investigation Molecular Core Laboratories using a commercially available pregnenolone Enzyme-Linked Immunosorbent Assay (ELISA) kit (Eagle Biosciences, Inc., Nashua, NH, USA). The ELISA kit has a sensitivity of 0.05 ng/mL, and a 100% specificity for pregnenolone. The coefficients of variation (CV) of intra-assay and inter-assay were < 10.6% and < 14.5%, respectively. For this ELISA, the cross reactivity between pregnenolone and other steroids was 6% for progesterone, 4.7% for 5alpha-androstanediol, 0.4% for pregnenolone sulfate, 0.3% for androstanedione, 0.2% for DHEAS, and less than 0.1% for several other steroids (e.g., androsterone, aldosterone, androstenedione, cholesterol, corticosterone, 5alpha-DHT, 17beta-estradiol, testosterone).

### 2.7. Data and Statistical Analysis

All statistical analyses were performed using SPSS software (SPSS Inc., Version 26, Chicago, IL, USA).Linear Mixed Effects (LME) models were used to assess craving, anxiety and autonomic responses, with within-subjects factors of Imagery Condition (stress, cocaine, neutral cues) andTimepoint (−20, −5, 0, +15, +30, +45, +60, +75), and Between-subjects factors of Medication Group (300 mg PREG, 500 mg PREG, placebo) as fixed effects. Subjects represented the random effect. LME models were also used to assess pregnenolone levels across imagery condition and timepoints (−20 and +75). LME models have the advantage of modeling the full response across timepoints and so if there is a missing data point, LMEs model the re-sponse across all timepoints and subjects and thus no subject is excluded due to miss-ing data [42]. Number of missing data collection at specific timepoints was low and its impact is minimized in LMEs. The Bonferroni test for multiple comparisons was used to analyze simple effects. Analysis of Variance (ANOVA) and Chi-square analyses were used to compare the medication groups on demographic variables. Figures were created with GraphPad Prism 9 (GraphPad Software Inc., San Diego, CA, USA). Any non-significant findings were not explicitly reported in the results. As significant main effects of Condition and Timepoint and significant interaction of Condition X Timepoint were expected, given our previous reports on validation of the experimental paradigm [3,36,38,42], these effects are not specifically reported here.

## 3. Results

### 3.1. Participant Characteristics, Balanced across the Groups

The medication groups did not significantly differ on any of the demographic characteristics (Table 1). About one third of the sample was female, and the sample was racially diverse with 53% of participants African American, 27% Caucasian, and 20% Hispanic. Importantly, race distributions were not significantly different across the groups. The average age was 48.5 years, majority of the sample consisted of regular nicotine smokers (7 non-smokers, 23 smokers), and the prevalence of regular smoking was balanced across the groups and did not differ significantly. The sample reported a long history of regular cocaine use, which was balanced across the groups.

### 3.2. Exogenous Pregnenolone Increases Circulating Pregnenolone Levels

**Figure 2:**
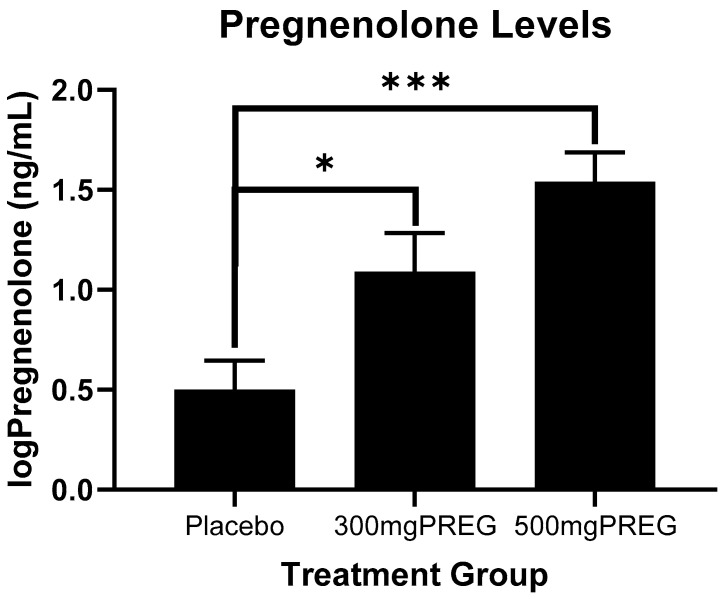
Log-transformed circulating pregnenolone levels as a function of placebo vs. PREG treatment groups, combined across timepoints and conditions. Pregnenolone levels were significantly higher in the 300 mg PREG group (*p* = 0.02) and the 500 mg PREG group (*p* = 0.0002) compared to placebo. All data are displayed as mean ± S.E.M. * *p* < 0.05; *** *p* < 0.001.

A main effect of medication group (F(2,15) = 12.6); *p* = 0.0001; f = 0.92) showed that circulating levels of pregnenolone were significantly higher in the 300 mg PREG group (*p* = 0.02) and the 500 mg PREG group (*p* = 0.0002) compared to the placebo group, and did not differ significantly between the PREG groups (*p* = 0.08) (Figure 2). There were not significant main effects of condition (*p* = 0.22) or timepoint (*p* = 0.97).

### 3.3. Pregnenolone Reduces Stress- and Cocaine Cue-Induced Cocaine Craving

**Figure 3:**
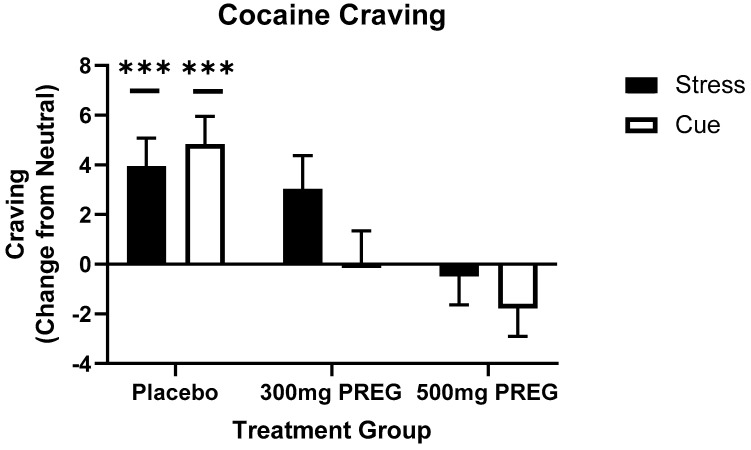
Provoked cocaine craving as a function of imagery condition and placebo vs. PREG treatment groups. Cocaine craving was significantly higher in the stress condition (*p* = 0.001) and the cocaine cue condition (*p* < 0.001) compared to the neutral condition in the placebo group. In the 300 mg PREG and 500 mg PREG groups stress- and cocaine cue-provoked craving was not significantly different than neutral (*p* = n.s.). All data are displayed as mean ± S.E.M. *** *p* < 0.001.

A main effect of condition (F(2,595) = 4.8; *p* = 0.008; f = 0.56) showed that cocaine craving was higher in the stress compared to the neutral condition (*p* = 0.006) across all three groups. However, a medication group x condition interaction (F(4,595) = 5.3; *p* < 0.001; f = 0.84) revealed that the PBO group had significantly higher cocaine craving in the stress condition (*p* = 0.001) and the cocaine cue condition (*p* < 0.001) compared to neutral, while the 300 mg and 500 mg PREG groups did not show such increases in stress and cue-provoked craving compared to the neutral condition (*p* = n.s.), see Figure 3. We also assessed whether pregnenolone levels correlated with craving, however this correlation was not significant.

### 3.4. Pregnenolone Dose-Specifically Reduces Stress- and Cocaine Cue-Anxiety

**Figure 4:**
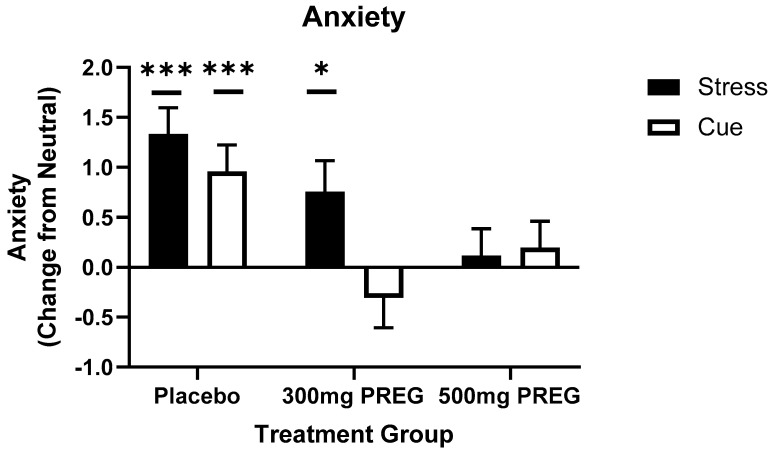
Anxiety as a function of imagery condition and placebo vs. PREG treatment groups. Stress-induced anxiety was significantly higher in the 300 mg PREG group compared to neutral (p = 0.016), and in the placebo group in response to stress (*p* < 0.001) and cocaine cue (*p* < 0.001) compared to neutral, but not in the 500 mg PREG group (*p* = n.s.). All data are displayed as mean ± S.E.M. * Significance represents condition relative to neutral. * *p* < 0.05; *** *p* < 0.001.

A main effect of condition (F(2,581) = 10.4; *p* < 0.001; f = 0.83) showed that anxiety was higher in the stress compared to the neutral (*p* < 0.001) and cocaine cue condition (*p* = 0.016) across all three groups. However, a medication group × condition interaction (F(4,581) = 4.7; *p* < 0.001; f = 0.79) showed that stress-induced anxiety was higher in the 300 mg PREG group compared to neutral (*p* = 0.04), and in the PBO group in the stress condition (*p* < 0.001) and the cocaine cue condition (*p* < 0.001) compared to neutral, whereas the 500 mg PREG group did not experience such stress- and cocaine cue-induced increases in anxiety (*p* = n.s.), see Figure 4.

### 3.5. Pregnenolone Dose-Specifically Reduces Autonomic Arousal

#### 3.5.1. Pregnenolone Reduces Stress-Induced Heart Rate (HR) Response in the 500 mg PREG Group

**Figure 5:**
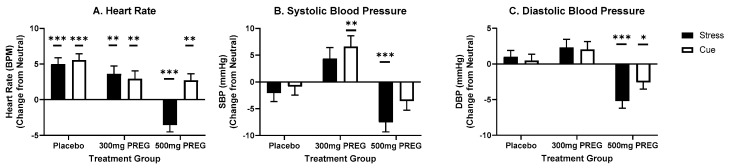
Pregnenolone effects on heart rate, systolic blood pressure and diastolic blood pressure. (**A**) Heart rate (HR) increased in response to stress and cocaine cue compared to the neutral condition in both the PBO group (S > N, *p* < 0.001; C > N, *p* < 0.001) and the 300 mg PREG group (S > N, *p* = 0.003; C > N, *p* = 0.02) and in response to cocaine cue in the 500 mg PREG group (*p* = 0.09), while it was significantly reduced in the stress condition compared to the neutral condition (*p* < 0.001) in the 500 mg PREG group. (**B**) Systolic blood pressure (SBP) increased significantly in response to the cocaine cue condition compared to the neutral condition (*p* = 0.003) in the 300 mg PREG group while it decreased significantly in the stress compared to neutral condition (*p* < 0.001) in the 500 mg PREG group. (**C**) Diastolic blood pressure (DBP) was significantly decreased in the stress condition (*p* < 0.001) and cocaine cue condition (*p* = 0.02) compared to neutral in the 500 mg PREG group. All data are displayed as mean ± S.E.M. * Significance represents condition relative to neutral. * *p* < 0.05; ** *p* < 0.01; *** *p* < 0.001.

A main effect of condition (F(2,558) = 23.0; *p* < 0.001; f = 1.24) showed that HR was significantly higher in the stress (*p* = 0.009) and cocaine cue condition (*p* < 0.001) compared to the neutral condition. A medication group x condition interaction (F(4,558) = 13.4; *p* < 0.001; f = 1.34) was observed, arising from significant increases in HR in response to stress compared to the neutral condition in the PBO group (*p* < 0.001) and the 300 mg PREG group (*p* = 0.003) while the 500 mg PREG group experience significant reduction in HR in the stress condition compared to the neutral condition (*p* < 0.001). In response to cocaine cue, higher increases in HR were observed compared to the neutral condition in both the PBO (*p* < 0.001), the 300 mg PREG group (*p* = 0.02), and in the 500 mg PREG group (*p* = 0.09), see Figure 5A.

#### 3.5.2. Pregnenolone Reduces Stress-Induced Systolic Blood Pressure (SBP) Response in the 500 mg PREG Group

A medication group x condition interaction (F(4,558) = 6.0; *p* < 0.001; f = 0.89) showed that the 300 mg PREG group had higher increases in mean systolic blood pressure (SBP) in the cocaine cue condition (*p* = 0.003) compared to the neutral condition. On the other hand, the 500 mg PREG group had a significant reduction in SBP in the stress condition (*p* < 0.001) compared to the neutral condition, while the PBO group had no condition interactions, see Figure 5B.

#### 3.5.3. Pregnenolone Reduces Stress and Cue-Induced Diastolic Blood Pressure (DBP) Response in the 500 mg PREG Group

A medication group x condition interaction (F(4,557) = 8.1; *p* < 0.001; f = 1.04) showed that the 500 mg PREG had significant reductions in DBP in response to the stress condition (*p* < 0.001) and the cocaine cue condition (*p* = 0.02) compared to neutral, while no such reductions were seen in either the PBO or the 300 mg PREG groups, see Figure 5C.

## 4. Discussion

This is the first study to test the effects of two supraphysiologic doses of pregnenolone in an established and validated model of provoked stress- and cocaine cue-induced craving, anxiety and autonomic arousal in individuals with cocaine use disorder (CUD). The study findings show that exogenous pregnenolone (a) increased circulating levels of pregnenolone, (b) decreased stress- and cue-induced craving, (c) dose-specifically reduced anxiety and (d) decreased autonomic responses at the higher dose but not the lower dose. Moreover, despite the small sample sizes and preliminary nature of the study, the results produced large effect sizes in the range of 0.5 to 1.34 for craving, anxiety, and autonomic measures indicating robust effect of pregnenolone doses. These effects sizes also indicated adequate power ranging from 0.90 to 0.99 with the current sample size for detecting differences between the tested doses of pregnenolone and placebo. The current results support the use of human laboratory models as an experimental therapeutic strategy for early assessment of the effects of target compounds such as pregnenolone for intermediate outcomes of drug craving, anxiety and physiological adaptations associated with CUD. On the basis of these results, current findings support pregnenolone as a promising target to reduce provoked craving, anxiety and autonomic system activation in CUD.

Both doses of pregnenolone significantly reduced stress- and cocaine cue-induced craving in this study. This finding is very important because, research has repeatedly shown that individuals with CUD experience a stress- and cocaine cue-induced craving state, which is accompanied by enhanced negative emotion and anxiety [3,38,43], and is a strong predicter of relapse [4]. While clinical research in CUD that directly targets neuroactive steroid potentiation is lacking, we previously reported that potentiation of allopregnanolone by high dose exogenous progesterone in individuals with co-morbid alcohol and cocaine use disorder normalized HPA axis responding to stress, improved cognitive performance in response to stress and cue, and overall decreased craving in the laboratory [44]. However, the current study is the first that assessed and found beneficial effects of pregnenolone administration on stress- and cocaine cue-induced craving in individuals with CUD.

Pregnenolone reduced cocaine-cue induced anxiety at both doses, and dose-specifically reduced stress-induced anxiety at the higher dose, but not at the lower dose. The reduction in anxiety is clinically relevant, as anxiety is one of the hallmark features of protracted early abstinence symptoms in cocaine use disorder [45,46], and exposure to stress- and cocaine cues results in significant increases in subjective anxiety in individuals with cocaine use disorder [3]. Therefore, reduction in anxiety should be an important target of effective treatment interventions for CUD. Neuroactive steroids have potent anxiolytic properties similar to those induced by other GABA_A_ receptor potentiating drugs [15,16]. Consistent with this previous research, current findings show stress- and cue-induced increases in anxiety in the placebo group, and the ability of the 500 mg/day of pregnenolone to reduce this sensitized anxiety response to stress may have tangible clinical relevance in the overall beneficial effects on treatment outcome and relapse risk in CUD.

Pregnenolone also had marked effects on autonomic responses to stress- and cocaine cue provocation. Pregnenolone at the higher dose, but not the lower dose, decreased stress-induced HR response. Similarly, the higher pregnenolone dose reduced stress-induced SBP, and stress-and cue-induced DBP. This is an important finding, as individuals with CUD consistently show adaptations in the autonomic nervous system [47]. Exposure to stress in individuals with cocaine use disorder results in significant increases in cardiovascular output, such as stress-induced increase in heart rate, and systolic and diastolic blood pressure [3]. Current findings suggest that pregnenolone may be reversing some of these autonomic adaptations and reducing the stress-induced cardiovascular hyperactivation, which was again observed in the placebo group.

The neuroactive steroid system is one of the most potent neuroendocrine pathways that plays a critical role in many vital functions of the human body, including direct modulation of GABAergic neurotransmission [16] and regulation of the physiologic arousal response to stress [9,10,11,12]. There is also mounting preclinical evidence that neuroactive steroids may play a role in reducing relapse to cocaine use in rats [12,31,32,33], confirming our findings observed here in a clinical population of individuals with CUD. We have also previously observed that chronic cocaine use is associated with decreased endogenous levels of pregnenolone [48]. These earlier studies, combined with the current findings, suggest that potentiation of the neuroactive steroid system in individuals with cocaine use disorder might offer a promising mechanism to normalize some of the adaptations that occur with chronic cocaine use and potentially reduce relapse risk.

The present study has a number of strengths. The study utilized a well-controlled and validated human laboratory experimental design to assess effects of stress and cocaine cue reactivity in a double-blinded, placebo controlled, dose-ranging study of pregnenolone. We also tested these effects of pregnenolone on a racially diverse population. Limitations of the study include a small sample size and recruitment of low numbers of women that prevented us from assessing sex differences. Future studies should also assess pregnenolone effects in non-CUD individuals. Additionally, this study only assessed pregnenolone as a single neuroactive steroid using an ELISA kit, but an overall picture of the pattern of downstream neurosteroid pathway, including allopregnanolone, in response to exogenous pregnenolone administration would be very important and informative. Moreover, in this study we only measured plasma levels of pregnenolone and not cerebrospinal fluid (CSF) or brain levels, which have been shown in animal models to not necessarily reflect each other. In addition, we only assessed pregnenolone levels at two timepoints: before imagery and at the end of the experimental procedure. It would be important for future studies to assess changes in pregnenolone levels and that of its downstream metabolites at various timepoints after provocation. Despite these limitations, this is the first clinical laboratory study to show preliminary efficacy of two doses of pregnenolone in reducing cocaine craving and anxiety, and positive effects in normalizing autonomic responses to stress and cocaine provocation in individuals with cocaine use disorder. Findings support the further development and investigation of these pregnenolone doses in evaluating potential therapeutic benefit in reducing cocaine use treatment outcomes.

## Figures and Tables

**Table 1:** Demographic Characteristics of the Sample.

	Placebo (*N* = 11)	300 mg PREG (*N* = 8)	500 mg PREG (*N* = 11)
Gender (Female [%])	3 [27.3]	3 [37.5]	3 [27.3]
Race Caucasian [%]	6 [54.5]	0 [0.0]	2 [18.2]
African American [%]	3 [27.3]	7 [87.5]	6 [54.5]
Hispanic [%]	2 [18.2]	1 [12.5]	3 [27.3]
Age (±SD)	46.9 (±9.2)	48.5 (±9.5)	50 (±7.8)
Years of Education (±SD)	12.6 (±2.0)	12.5 (±2.0)	12.6 (±2.3)
No. of Regular Smokers [%]	10 [90.9]	4 [50.0]	9 [81.8]
Years of Cocaine Use (±SD)	14.7 (±7.9)	23.8 (±10.4)	15.3 (±13.4)

All variables: *p* > 0.05.

## Data Availability

Not applicable.
